# Supplementary material for: Genome-wide identification and characterization of FORMIN gene family in potato (Solanum tuberosum L.) and their expression profiles in response to drought stress condition
Source: PLoS One. 2024 Aug 26;19(8):e0309353. doi: 10.1371/journal.pone.0309353 (PMC11346945; doi:10.1371/journal.pone.0309353)
Supplement: S6 Data — (DOCX) [file pone.0309353.s006.docx]

**S6 Data.** StFH protein family distribution among groups based on phylogenetic analysis with *Zea mays, Oryza sativa, Arabidopsis thaliana, Medicago truncatula* and *Lotus japonicas* FH2 members.

| Group | Number of StFH proteins | StFH protein family members |
| --- | --- | --- |
| A | 3 | *StFH5, StFH8, StFH18* |
| B | 2 | *StFH10, StFH26* |
| C | 2 | *StFH4, StFH6* |
| D | 12 | *StFH7, StFH11, STFH12, StFH13, StFH14, StFH15, StFH16, StFH20,*  *StFH21, StFH22, StFH23, StFH24,* |
| E | 1 | *StFH1* |
| F | 2 | *StFH2, StFH3* |
| G | 4 | *StFH9, StFH17, StFH19, StFH25* |
