# Supplementary material for: Genome-wide identification and characterization of FORMIN gene family in potato (Solanum tuberosum L.) and their expression profiles in response to drought stress condition
Source: PLoS One. 2024 Aug 26;19(8):e0309353. doi: 10.1371/journal.pone.0309353 (PMC11346945; doi:10.1371/journal.pone.0309353)
Supplement: S7 Data — (DOCX) [file pone.0309353.s007.docx]

**S7 Data.** *In silico* predicted the number of introns and exons in *StFH* genes.

| **Group name** | **Gene name** | **Source accession** | **Introns** | **Exons** |
| --- | --- | --- | --- | --- |
| A | *StFH5* | Soltu.DM.03G029790 | 16 | 17 |
|  | *StFH8* | Soltu.DM.06G025740 | 16 | 17 |
|  | *StFH18* | Soltu.DM.08G021110 | 16 | 17 |
| B | *StFH10* | Soltu.DM.07G000540 | 16 | 17 |
|  | *StFH26* | Soltu.DM.12G027420 | 18 | 19 |
| C | *StFH4* | Soltu.DM.03G027630 | 1 | 2 |
|  | *StFH6* | Soltu.DM.05G007060 | 1 | 2 |
| D | *StFH7* | Soltu.DM.05G018890 | 5 | 6 |
|  | *StFH11* | Soltu.DM.07G018190 | 7 | 8 |
|  | *StFH12* | Soltu.DM.07G018220 | 2 | 3 |
|  | *StFH13* | Soltu.DM.07G018270 | 1 | 2 |
|  | *StFH14* | Soltu.DM.07G018320 | 4 | 5 |
|  | *StFH15* | Soltu.DM.07G018330 | 5 | 6 |
|  | *StFH16* | Soltu.DM.07G025400 | 4 | 5 |
|  | *StFH20* | Soltu.DM.10G001740 | 5 | 6 |
|  | *StFH21* | Soltu.DM.10G001770 | 3 | 4 |
|  | *StFH22* | Soltu.DM.10G001820 | 2 | 3 |
|  | *StFH23* | Soltu.DM.10G001850 | 4 | 5 |
|  | *StFH24* | Soltu.DM.10G001880 | 5 | 6 |
| E | *StFH1* | Soltu.DM.01G039360 | 3 | 4 |
| F | *StFH2* | Soltu.DM.02G027760 | 3 | 4 |
|  | *StFH3* | Soltu.DM.03G005470 | 4 | 5 |
| G | *StFH9* | Soltu.DM.06G033180 | 3 | 4 |
|  | *StFH17* | Soltu.DM.08G006050 | 3 | 4 |
|  | *StFH19* | Soltu.DM.09G015150 | 3 | 4 |
|  | *StFH25* | Soltu.DM.12G019570 | 3 | 4 |
