# Supplementary figures and images for: Genome-wide identification and characterization of FORMIN gene family in potato (Solanum tuberosum L.) and their expression profiles in response to drought stress condition
Source: PLoS One. 2024 Aug 26;19(8):e0309353. doi: 10.1371/journal.pone.0309353 (PMC11346945; doi:10.1371/journal.pone.0309353)

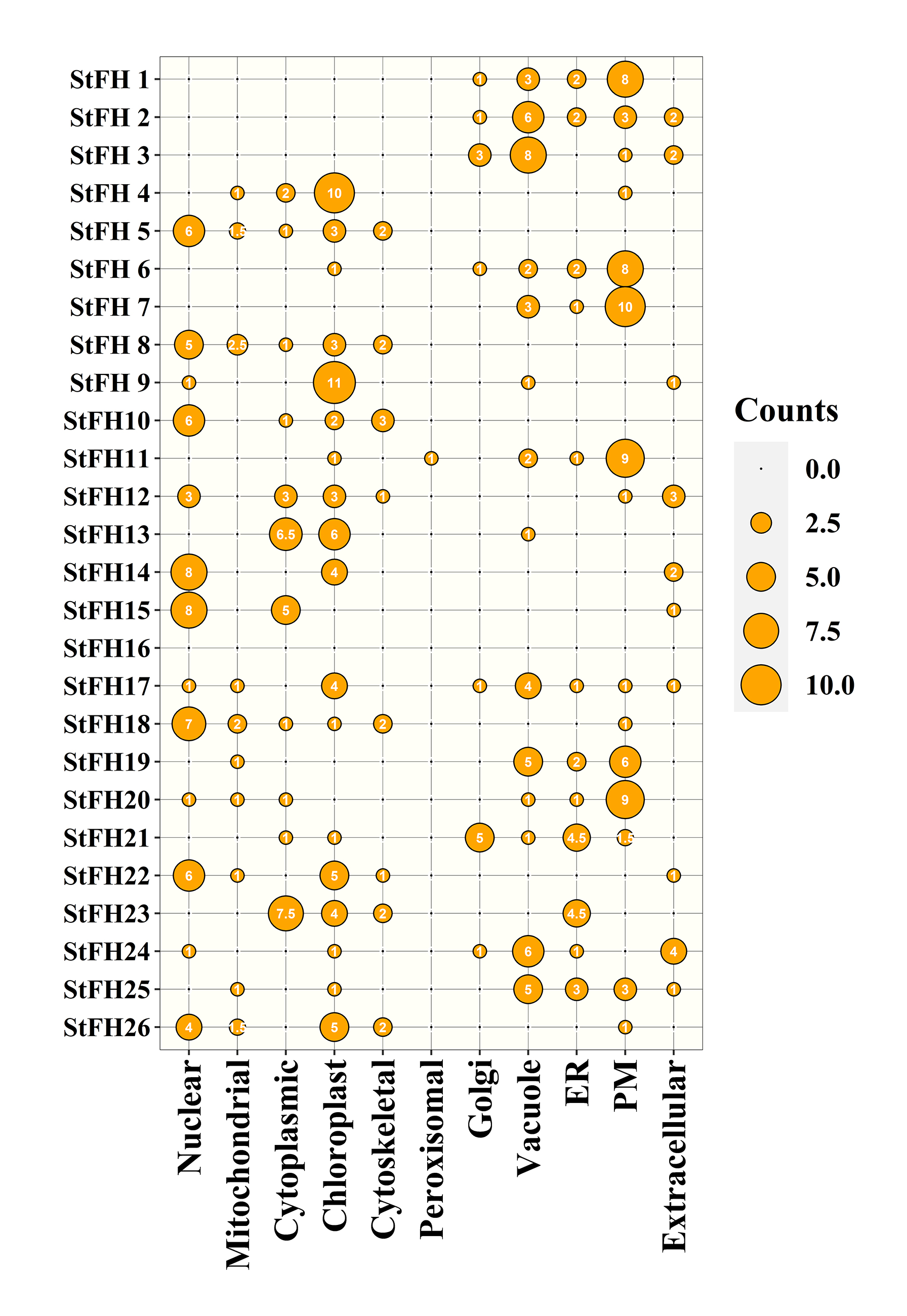

Supplement: S1 Fig — (TIFF) [file pone.0309353.s015.tiff]
