# Supplementary material for: Genome-wide identification and characterization of FORMIN gene family in potato (Solanum tuberosum L.) and their expression profiles in response to drought stress condition
Source: PLoS One. 2024 Aug 26;19(8):e0309353. doi: 10.1371/journal.pone.0309353 (PMC11346945; doi:10.1371/journal.pone.0309353)
Supplement: Attachment — Submitted filename: reviwer report.docx [file pone.0309353.s016.docx]

The paper titled: " Genome-wide identification and characterization of FORMIN gene family in potato (Solanum tuberosum L.) and their expression profiles in response to drought stress condition " reported the identification, characterization and of FORMIN gene family in Solanum tuberosum and the expression patterns of *FORMIN* members using RNA-seq data, offering a foundation for further research aiming at potato enhancement. Overall, the paper is clearly presented. The authors are invited to revise the English and the sentences structure in the manuscript before publication. Also, genes must be written in Italic in the whole text.
