# Supplementary material for: Genome-wide identification and characterization of FORMIN gene family in potato (Solanum tuberosum L.) and their expression profiles in response to drought stress condition
Source: PLoS One. 2024 Aug 26;19(8):e0309353. doi: 10.1371/journal.pone.0309353 (PMC11346945; doi:10.1371/journal.pone.0309353)
Supplement: Attachment — Submitted filename: Response to Reviewers.docx [file pone.0309353.s017.docx]

**Response to Reviewers**

**Reviewers' comments:**
**Responses to Reviewer's Questions**

**Review Comments to the Author**

**Reviewer #1:**

In this manuscript, authors identified a total of 26 StFH genes in the *S. tuberosum* genome, and analyzed their expression profiling, subcellular location, duplication and selection pattern, miRNA targeting prediction, and protein-protein interaction. However, there are a few points which should be improved, as delineated below.

**Comment #1:** The well-crafted professional editing service should be employed for this manuscript. Additionally, it is necessary to reduce the number of words in this Abstract, owing to 300-word limit in the PlosONE’s Abstract.

**Responses to Comment #1:** Thank you for your valuable suggestion to improve our manuscript for language usage, spelling, and grammar by getting English editing and proofreading services. We are pleased to inform you that, to improve our manuscript at a standard level and to avoid grammatical errors, we are thoroughly edited and proofreading of our full manuscript by an academic English professional named Mr. Tanzir Ahmed, Assistant Professor, Department of English, Faculty of Arts and Social Science, Jashore University of Science and Technology, Jashore 7408, Bangladesh (<https://just.edu.bd/t/teacher-1549444621686>), email: [tanzir@just.edu.bd](mailto:tanzir@just.edu.bd) which are highlighted as a track change options (Please see the track changes area around the full manuscript). We also acknowledged his name in the acknowledgement section of our manuscript (Please see the lines 833-836). Hopefully, the language of the revised manuscript is now more standard than the previous one.

Moreover, we have reduced the word number below 300 limits to PlosONE’s Abstract style.

**Comment #2:** The authors should provide tissue/organ-specific and drought-responsive expression pattern of StFH genes using RT-qPCR.

**Responses to Comment #2:** Thank you for your valuable comment and suggestion. We appreciate your insights and fully agree with the importance of validating the candidate *StFH* genes using RT-qPCR through wet lab experiments. We would like to inform you that, currently we do not have a enough research grant to support the experimental validation of the data in our laboratory or to seek assistance from other well-equipped facilities. We sincerely apologize for the inconvenience caused regarding RT-qPCR experiment and hope for your understanding and consideration regarding this issue.

**Comment #3:** To further validate the predicted results, it is necessary to provide experimental evidence for the subcellular localization of StFHs.

**Responses to Comment #3:** Thank you for your valuable necessary comment and suggestion. We also agree with the importance of provide experimental evidence for the subcellular localization of StFHs experimental evidence for the subcellular localization of StFHs through wet lab experiments. We cordially would like to inform you that, currently we do not have a research grant to support the experimental validation of the data in our laboratory or to seek assistance from other well-equipped facilities. We sincerely apologize for the inconvenience caused regarding experimental evidence for the subcellular localization of StFHs and hope for your understanding and consideration regarding this issue.

**Comment #4:** The version of tools, such as BlastP (Line121), MEGA11 (L139), ClustalW (L140), Trimmonmatic (L231), bowtie2 (L234), Samtools (L235) and RSEM (L236), should be replenished in the ‘Materials and Methods’ section.

**Responses to Comment #4:** Thank you very much for your kind suggestions. According to your comment, we have replaced the above-mentioned tools explanation in the ‘Materials and Methods’ section of our revised manuscript. Please see the revised section of ‘Materials and Methods’ and lines 119-124, and lines 208-214.

**Comment #5:** The splice-aware mappers (e.g., TopHat2/HISAT2, STAR) are capable of mapping RNA-seq reads against the reference genome. However, the Bowtie2 is not splice-aware aligner, and used frequently for the mapping of DNA-seq reads to the reference genome. Bowtie2, therefore, is not suitable for alignment of RNA-seq reads to the *S. tuberosum genome* (L234).

**Responses to Comment #5:** Thank you for your valuable comment on alignment of RNA-seq reads to the *S. tuberosum genome* for expression pattern analysis of *StFH* genes in drought stress treatments. According to your comment, we have redesign our RNA-seq reads pipeline based on STAR splice-aware aligner and used this STAR aligner for mapping the raw reads of of RNA-seq reads to the *S. tuberosum genome*. Surprisingly, we have got excellent results were using the STAR splice-aware aligner for mapping the raw reads of of RNA-seq reads to the *S. tuberosum genome*. We are very pleased to your kind and valuable suggestions and comments regarding splice-aware mappers (e.g., TopHat2/HISAT2, STAR). We also acknowledge your great suggestions which was unknown to us. Next time, we will use these splice-aware mappers. According to the new analysis results, we have revised the methods and materials as well as results and discussion section of expression pattern analysis of *StFH* genes. Please see the revised Fig. 16 and lines 208-214.

**Comment #6:** In Fig.1, the bootstrapping value should be added in the branch of the maximum likelihood (ML) tree. Compared to MEGA, IQ-TREE or RAxML might be a more appropriate tool for reconstructing ML tree, especially in the choice of the best-fitting substitution model.

**Responses to Comment #6:** Thank you for your valuable comment on Fig. 1. According to your comment, we have used the IQ tree version 1.6.12 with default parameters (<http://www.iqtree.org/>) to construct the phylogenetic tree. Also, we have added the bootstrapping value in the branch of the maximum likelihood (ML) tree according to your kind comment. According to the new analysis results, we have revised the methods and materials as well as results and discussion section of phylogenetic tree analysis. Please see the revised Fig. 1 and lines120-124,

**Comment #7:** In Fig.5, StFH20 and StFH22 were identified as a duplicated gene pair. It might be problem, owing to a great difference in their CDS length (Fig.3, > 3 fold). DupGen_finder or MCScanX should be a toolkit for detection of duplicated gene pairs.

**Responses to Comment #7:** Thank you very much for your kind and valuable comment on Fig. 5. According to your comment, we have used MCScanX toolkit for detection of duplicated gene pairs. Surprisingly, we have got good results by using MCScanX toolkit instead of our used previous analysis technique. Again, thanks a lot for providing us an incredible valuable suggestion and comment for the analysis of duplicated gene pairs. According to the new analysis results, we have revised the results and discussion section of Synonymous (Ks) and non-synonymous (Ka) substitution ratios calculation of *StFH* genes. Please see the revised Fig. 5 and line 145-146.

**Comment #8:** The gene name should be italicized, e.g., StFH (Line36, L40, L848, L851, L852, L854).

**Responses to Comment #8:** Thank you so much for your valuable comment. According to your comment, we have italicized all gene names in the whole manuscript.

**Reviewer #2:**

The paper titled: " Genome-wide identification and characterization of FORMIN gene family in potato (Solanum tuberosum L.) and their expression profiles in response to drought stress condition " reported the identification, characterization and of FORMIN gene family in Solanum tuberosum and the expression patterns of FORMIN members using RNA-seq data, offering a foundation for further research aiming at potato enhancement. Overall, the paper is clearly presented. The authors are invited to revise the English and the sentences structure in the manuscript before publication. Also, genes must be written in Italic in the whole text.

**Comment #1:** The authors are invited to revise the English and the sentences structure in the manuscript before publication. Also, genes must be written in Italic in the whole text.

**Responses to Comment #1:** Thank you for your valuable suggestion to improve our manuscript for language usage, spelling, and grammar by getting English editing and proofreading services. We are pleased to inform you that, to improve our manuscript at a standard level and to avoid grammatical errors, we are thoroughly edited and proofreading of our full manuscript by an academic English professional named Mr. Tanzir Ahmed, Assistant Professor, Department of English, Faculty of Arts and Social Science, Jashore University of Science and Technology, Jashore 7408, Bangladesh (<https://just.edu.bd/t/teacher-1549444621686>), email: [tanzir@just.edu.bd](mailto:tanzir@just.edu.bd) which are highlighted as a track change options (Please see the track changes area around the full manuscript). We also acknowledged his name in the acknowledgement section of our manuscript (Please see the lines 833-836). Hopefully, the language of the revised manuscript is now more standard than the previous one. Moreover, according to your valuable comment, we have carefully checked and italicized all gene names in the whole manuscript.
