# Supplementary material for: Genome-wide identification and characterization of FORMIN gene family in potato (Solanum tuberosum L.) and their expression profiles in response to drought stress condition
Source: PLoS One. 2024 Aug 26;19(8):e0309353. doi: 10.1371/journal.pone.0309353 (PMC11346945; doi:10.1371/journal.pone.0309353)
Supplement: Attachment — Submitted filename: Response to Reviewers.docx [file pone.0309353.s018.docx]

**Response to Reviewers**

**Reviewers' comments:**
**Responses to Reviewer's Questions**

**Review Comments to the Author**

**Reviewer #1:**

**Comment #1:** Frist of all, thanks to the authors for their efforts in revising this manuscript. However, it is very unfortunate to know that these experiments (Comment #2, #3) could not be carried out due to lack of funds.

Please add literature (Line150).

**Responses to Comment #1:** Thank you for your valuable suggestion and consideration for your previous comments (Comment #2, #3) regarding our limitation in wet lab validation due to our lack of research funds. In this revision, according to your suggestion, we have added the literature in Line 150. Please see the Line150.

**Reviewer #2:**

**Comment #1:** The quality of the manuscript is clearly more better with this revision but a number of english errors are still detectible in the text. the text must be throughly revised to be free of english mistakes.

**Responses to Comment #1:** Thank you for your valuable suggestion to improve our manuscript avoiding language grammar errors, and common mistakes. According to your kind suggestions, in this revision, we have vigorously revised our manuscript to avoid grammatical errors, and common mistakes as well as tried to improve our manuscript at a standard level. In this revision, we also take the English language editing and proofreading opportunity from our academic English professional named Mr. Tanzir Ahmed, Assistant Professor, Department of English, Faculty of Arts and Social Science, Jashore University of Science and Technology, Jashore 7408, Bangladesh (<https://just.edu.bd/t/teacher-1549444621686>), email: [tanzir@just.edu.bd](mailto:tanzir@just.edu.bd) which are highlighted as a track change options (Please see the track changes area around the full manuscript). We also acknowledged his name in the acknowledgment section of our manuscript (Please see lines 766-769). Hopefully, the language of the revised manuscript is now more standard than the previous one and you will consider it.
