# Supplementary material for: Genome-wide identification and characterization of FORMIN gene family in potato (Solanum tuberosum L.) and their expression profiles in response to drought stress condition
Source: PLoS One. 2024 Aug 26;19(8):e0309353. doi: 10.1371/journal.pone.0309353 (PMC11346945; doi:10.1371/journal.pone.0309353)
Supplement: Attachment — Submitted filename: Response to Reviewers.docx [file pone.0309353.s019.docx]

**Response to Reviewers**

**Reviewers' comments:**
**Responses to Reviewer's Questions**

**Reviewer#1:**

**Comment#1:** The expression pattern of FORMIN genes in Solanum tuberosum under drought stress is a main content of this manuscript entitled ‘Genome-wide identification and characterization of FORMIN gene family in potato (Solanum tuberosum L.) and their expression profiles in response to drought stress condition’. In addition, RT-qPCR results have been provided in a few recently published papers, such as Zhang et al. (PMID: 38256829) and Duan et al. (PMID: 34303338). Thus, it is necessary to provide the tissue/organ-specific and drought-responsive expression pattern of StFH genes using RT-qPCR.

**Responses to Comment#1:** Thank you for your valuable comment. According to your valuable comment, we have extracted two RNA-seq data for tissue/organ-specific and drought-responsive expression patterns of *StFH* genes. We have revised our manuscript based on those two data sets. Please see the details for tissue/organ-specific and drought-responsive expression patterns of *StFH* genes in the Materials and Methods section (Lines 200-220), Results section (Lines 500-535), Discussion section (Lines 642-666), Abstract section (Lines 43-47), Conclusion section (Lines 678-682). Thank you so much for providing the recent research articles related to expression analysis of this gene family members in other plant species.

In Review-1, Comment#2 of your Response to Comment#2, as we previously mentioned to you, we always appreciate your insights and fully agree with the importance of validating the candidate *StFH* genes using RT-qPCR through wet lab experiments. We would like to inform you that, currently we do not have a enough research grant to support the experimental validation of the data in our laboratory or to seek assistance from other well-equipped facilities. We sincerely apologize for the inconvenience caused regarding the RT-qPCR experiment and hope for your understanding and consideration regarding this issue.

**Comment#2:** Solanum tuberosum is composed of two subspecies, such as tuberosum and andigena. There are a few potato genomes available at SpudDB (http://spuddb.uga.edu/), such as S. tuberosum cv. Otava (autotetraploid), S. tuberosum Group Phureja DM 1-3 516 R44 (doubled monoploid), and S. tuberosum group Tuberosum RH89-039-16 (heterozygous diploid). Thus, it is very fascinating how StFH genes are distributed on multiple different subgenomes of S. tuberosum.

**Responses to Comment#2:** Thank you for your valuable comment. According to your nice and valuable comment, we have retrieved the genome information of three cultivars of *Solanum tuberosum*, *S. tuberosum* cv. Otava (autotetraploid), *S. tuberosum* Group Phureja DM 1-3 516 R44 (doubled monoploid), and *S. tuberosum* group Tuberosum RH89-039-16 (heterozygous diploid) from SpudDB (<http://spuddb.uga.edu/>) database and constructed a chromosomal distribution of *StFH* genes within the above three subgenomes of *S. tuberosum*. Hopefully, this *StFH* gene distribution information will enhance our manuscript quality. We will be grateful to you for your valuable comment on this. Please see the Fig 9 and Lines 157-163, 353-365, and 577-580. Again, thank you for your kind suggestion and provided us the important link.

**Comment#3:** It is prerequisite to determine the syntenic relationship of FH genes in Solanum tuberosum with the other three species, including Arabidopsis thaliana, Oryza sativa and Zea may, such as the Figure 5 in the paper of Zhang et al. (PMID: 38256829). However, the Fig7 in this manuscript does not seem to reveal the collinear relationship of FH genes between Solanum tuberosum with the other three species. Thus, the interspecific synteny relationship of FH genes should be redrawn via TBtools (https://github.com/CJ-Chen/TBtools-II) or JCVI (https://github.com/tanghaibao/jcvi).

**Responses to Comment#3:** Thank you for your valuable and kind comment. According to your valuable comment, we have constructed an interspecific synteny relationship of *FH* genes within three species such as Arabidopsis thaliana, Oryza sativa, and Zea mays including Solanum tuberosum using MCScanX of TB tools v.2.010 (<https://github.com/CJ-Chen/TBtools-II>). We hope this information will also increase our manuscript quality. Thank you so much for your nice comment. Please see the Fig 7 and Lines 147-151, 321-334, and 587-589.

**Comment#4:** There are still a lot of writing errors. a) Writing errors in the Abstract section: ‘processes’ was falsely written as ‘processess’(Line26 ==26); ‘a genome-wide analysis of’ should be rewritten as ‘no genome-wide analysis of’ (Line27 ==L26); ‘CAREs’ should be rewritten as ‘cis-acting regulatory elements (CAREs)’ (Line36 ==L36); ‘uncovered’ was mistakenly written as ‘uncoverd’ (Line41 ==Excluded).

**Responses to Comment#4(a):** Thank you for your valuable comments and suggestions. We have corrected the mentioned lines according to your comments. The word ‘uncovered’ is replaced with ‘identified’. Please see the lines 26, 26, 36, and 42.

**Comment#4(b):** In the Line173, ‘PlantRegMap’ was irrelevantly written as ‘PlantTFDB’

**Responses to Comment#4(b):** Thank you for your valuable comment. We have corrected the term as ‘PlantRegMap’. Please see the lines 174-175.

**Comment#4(c):** In the Line179, a full stop was lost in the sentence ‘numerous important transcription factors (TFs). The collected data was subsequently visualized’.

**Responses to Comment#4(c):** Thank you for your valuable comment. We have corrected it. Please see the line 181.

**Comment#4(d):** In the Line490, ‘MicroRNAs’ was wrongly written as ‘MmicorRNAs’.

**Responses to Comment#4(d):** Thank you for your valuable comment. We have corrected the term. Please see the line 468.

**Comment#4(e):** The genus name should be italicized, such as Arabidopsis (Line506, Line524, Line672).

**Responses to Comment#4(e):** Thank you for your valuable comment. We have italicized the genus name, *Arabidopsis*. Please see lines 480, 494, and 620.

**Comment#4(f):** In the Line718, ‘across’ should be revised as ‘in’.

**Responses to Comment#4(f):** Thank you for your valuable comment. We have replaced ‘across’ with ‘in’. Please see the line 669.

**Reviewer #2:**

**Comment#1:** The authors of the paper titled "Genome-wide identification and characterization of FORMIN gene family in potato (Solanum tuberosum L.) and their expression profiles in response to drought stress condition" have correctly adressed the required revisions

**Responses to Comment#1:** Thank you so much for giving your valuable time, kind comments, and suggestions to improve the quality of our manuscript. We are also very pleased to address your all previous comments and suggestions.
